# Masticatory muscle activity evaluation by electromyography in subjects with zygomatic implants

**DOI:** 10.4317/medoral.21659

**Published:** 2017-04-08

**Authors:** Moara de Rossi, Marcelo Palinkas, Bárbara Lucas, Carla Santos, Marisa Semprini, Ligia Oliveira, Isabela Regalo, Edmilson Bersani, Reginaldo Migliorança, Selma Siéssere, Simone Regalo

**Affiliations:** 1 Department of Morphology, Physiology and Basic Pathology, School of Dentistry of Ribeirão Preto, University of São Paulo, Brazil

## Abstract

**Background:**

Zygomatic implants are an alternative treatment in the rehabilitation of atrophic maxilla to promote stability in the stomatognathic system. The aim of this study was to compare the electromyographic (EMG) activity of masseter and temporalis muscles in controls and in individuals with complete implant-supported dentures anchored in the zygomatic bone.

**Material and Methods:**

Fifty-four volunteers of both genders (mean age 52.5 years) were selected and distributed into two groups: Individuals with zygomatic implant (ZIG; n=27) and fully dentate patients (CG; n=27). MyoSystem-BR1 was used to assess masseter and temporalis muscles EMG activity in different mandibular movements: protrusion, clenching, maximal voluntary contraction (MVC) with Parafilm M®, right and left laterality and chewing (peanuts and raisins). Data was processed, normalized (MVC) and analyzed using the SPSS 21.0. Student t-test (*P* ≤ 0.05) was used for group comparison.

**Results:**

The results were statistically significant (*P* ≤ 0.05) for protrusion, clenching, right and left laterality and raisin chewing. For the mandibular posture conditions, the ZIG obtained higher EMG activity patterns when compared to CG. For the masticatory performance during chewing of peanuts and raisins, the ZIG showed higher EMG mean values when compared to CG.

**Conclusions:**

The zygomatic implant promoted an active response of the muscle fibers (hyperactivity) during both mandibular posture and chewing conditions, probably due to the absence of periodontal receptors, which play a significant role for preparing a bolus for swallowing.

** Key words:**Zygomatic implant, electromyography, masseter muscle, temporal muscle.

## Introduction

Dental science has been renewed with the osseointegration process that involves the biocompatibility of the implant material with bone. This interaction provides innovative dental treatment for complete or partial edentulous individuals and can reestablish the masticatory function, retention and stability of the fixed restorations (1), with satisfactory results for the stomatognathic system (2,3).

During chewing, mandible elevation and depression movements are centrally determined and modulated by receptors, which are found in periodontium, temporomandibular joints, tongue, oral mucosa, tendons, and muscle spindles of elevator muscles. Thus, all these oral structures play an important role in mastication and in longevity of the dentition (4). The loss of one or several dental elements brings clinical consequences that can not only affect the alveolar process, but also reduce the masticatory efficiency and sometimes result in nutritional deficiency (5), functional asymmetry of the masticatory muscles and temporomandibular disorders (6).

For edentulous or partially edentulous individuals, with limited functional conditions and severe bone loss, implants subjected to immediate functional loading are indicated to reduce the time interval between surgery and prosthesis fixation (7). A well planned and performed implant technique provides safe treatments that can significantly improve the neuromuscular function of the masticatory system, and therefore, the quality of life (8,9).

The zygomatic implants (10,11), provides support for the dental prosthesis to rehabilitate edentulous arches with substantial amount of bone loss. Surgical treatments that use these specific and different techniques aim to re-establish the oral function, to reduce the treatment time, to promote prosthetic retention, and to make an anatomical model of the mandible (12).

Thus, the aim of this study was to compare EMG activity of the masseter and temporalis muscles of dentate subjects and those with fully fixed prosthesis supported by the zygomatic implant not only during masticatory cycles of habitual mastication but also during mandibular posture conditions.

## Materials and Methods

This study was carried out at the “Prof. Dr. Mathias Vitti” Electromyography Laboratory, Department of Morphology, Physiology and Basic Pathology, University of São Paulo-USP. The research project was approved by the Research Ethics Committee and conducted in accordance with the Declaration of Helsink. Participants were informed about the experiment and agreed to participate by providing their free and informed consent according to resolution 466/12 of the Health National Council.

- Sample 

When calculating the sample size, the standard deviation of responses was increased to achieve a power sufficient to catch large effects with fewer subjects included. Under these conditions, a maximum level of significance α = 0.05 and a minimal test power of 80% were adopted, assuming a 30% sample loss, with n = 27 individuals in each group.

The sample and the inclusion and exclusion criteria were established by means of anamneses, clinical exams, and presence signs and symptoms of temporomandibular disorders (TMD) through specific questionnaire (Research Diagnostic Criteria for TMD, RDC/TMD) (13), which provided data regarding the participants’ personal information, medical and dental history, any existing parafunctional habits or signs and symptoms of temporomandibular dysfunction.

Subjects with satisfactory implant-supported prostheses using the zygomatic implant system for at least six months were included in the study (14). The subjects were submitted to a surgical technique to place the zygomatic implant anchorage platform closer to the alveolar ridge, without passing through the region of the maxillary sinus (10).

**Figure 1:**
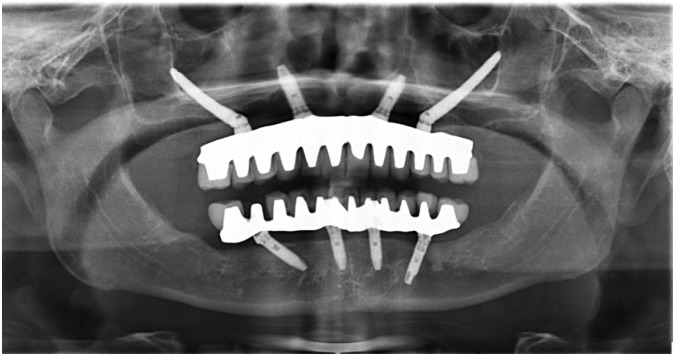
Panoramic radiograph of implants anchored in the zygomatic bone.

**Figure 2:**
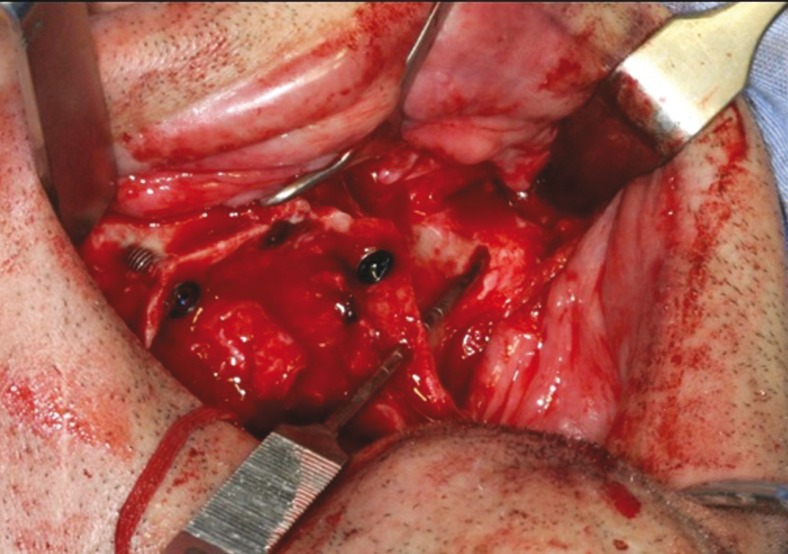
Surgical technique of zygomatic implants.

The surgical access was similar to that of Brånemark technique (2); however, with a different approach advocated by Reginaldo Migliorança, where bone drilling sequence is made completely outside the internal region of the maxillary sinus (Fig. 1). After the flap was reflected, the bone drilling began with a spherical drill, marking a trajectory of 2.9mm drill, which penetrated the residual alveolar ridge, emerging through the vestibular, external to the maxillary sinus, until it reached the zygomatic bone. The same drill surpassed the cancellous layer of the bone. With the use of a depth indicator, the length of the zygomatic implant was determined and defined as 2mm less than the obtained measurement. The osteotomy was widened using the following drills: twist drill 2.9, pilot drill 3.5 and twist drill 3.5, respectively. The final placement of the zygomatic implant was the closest possible to the top of the crest of the alveolar ridge, preferably in the first molar region (Fig. 2).

All conditions that could interfere with the muscle activity were used as exclusion criteria after the anamnesis interview: presence of dental pathology, oral and maxillofacial pain, systemic or local disorders (neurological disorders and cerebral palsy); use of unsatisfactory implant-supported prosthesis, or any medication.

**Figure 3:**
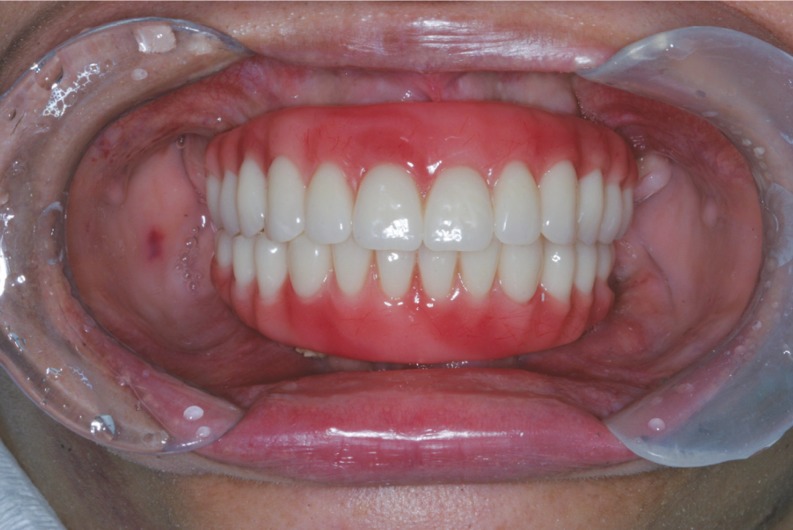
Rehabilitation pattern with at least 6 months of zygomatic implants.

Participants submitted to either orthodontic or otorhinolaryngology treatments as well as to speech therapy were also excluded. Fifty-four volunteers of both genders, between the ages of 35 and 70 years (mean age 52.5 years) were selected for this study and distributed into two groups: ZIG (Zygomatic Implant, n=27) and CG (fully dentate subjects n=27). The groups were matched subject to subject by gender, age and body mass index. The use of mandibular overdenture was considered the oral rehabilitation pattern for all ZIG subjects (Fig. 3).

- Electromyographic Analysis 

The EMG analysis was performed using the Myosystem BR1 apparatus (Data Hominis Ltd., Uberlandia, MG, Brazil) attached to a laptop, with 12-channels, including eight channels for EMG (active and passive electrodes) and four auxiliary channels. Surface active differential electrodes (two 10 mm-long x 2 mm-wide silver chloride bars 10 mm apart) with input impedance of 1010 Ω/6 pf, bias current input of ±2 nA, common-mode rejection ratio of 110 dB at 60 Hz and gain equal to 20x were used in the study.

The EMG signals were further amplified by 50x (total gain 1000x), bandpass filtered (20 Hz–1 kHz) and sampled at a frequency of 2 kHz with 16 bits resolution. The data were visualized and processed using the program Myosystem I (version 3.5), which enables the definition of processing windows and calculate various features, such as frequency spectrum, RMS and linear envelop. The reference electrode was positioned on the right wrist and the differential active electrodes were positioned in the ventral region of the masseter muscles and in the right and left portion of the temporalis muscles with the longest extension of the bars perpendicular to the direction of the muscle fibers.

The position of the electrodes was determined by palpation. Specific maneuvers of maximum voluntary contraction were carried out to ensure the precise location of the muscles (15). The electrodes were fixed with adhesive tape (Cremer SA; Blumenau, Santa Catarina, Brazil), allowing a total contact between the electrode and the skin.

The examination room was kept silent and peaceful throughout the recording sessions. The volunteers seated on a comfortable chair, the head in upright position, feet touching the ground, and the hands lying on their thighs. The head remained erect and the eyes aligned with the horizon. For the EMG evaluation, the masseter and temporalis muscles were analyzed the following clinical conditions: protrusion (10s), right laterality (10s), left laterality (10s); dental clenching (4s) and maximal voluntary contraction on twice-folded parafilm sheet (Parafilm M®, Pechinery Plastic Packaging, Batavia, IL, USA) (18x17x4mm, 245 mg) and positioned between the occlusal surfaces of the superior and inferior first molars, bilaterally.

For the dynamic evaluation of forces during mastication, the efficiency of the masticatory cycles was verified by means of the integrated linear envelope EMG of the masseter and temporalis muscles (bilaterally). The values were registered in units of (microvolt/seconds). The EMG signals were collected during habitual mastication of hard (5g of Mendorato® Japanese peanuts, Santa Helena S/A., Ribeirão Preto, São Paulo, Brazil) and soft foods (5g of raisins) for 10 seconds. Both natural foods were from the same batch and were kept in a cool, airy place, in individual plastic containers.

For this evaluation, the isometric contractions were verified by calculating the integral of the linear envelope of each muscle, from the moment the food exerted pressure between the teeth, or when there was occlusal contact, providing measurements of the area vs. time (16). The values of the electromyographic signals during mastication were obtained from the central masticatory cycles. First cycles vary considerably in the initial phase of the masticatory process (17), thus, the initial masticatory cycles were excluded. The EMG data were rectified before integrate the data (microvolts-second).

- Statistical Analysis 

Following the EMG data collection, the Shapiro-Wilk normality test was applied to determine the normality of the sample. Student’s t-test was applied for group comparison. The level of statistical significance was set at *P* ≤ 0.05 for a 95% confidence interval (SPSS Inc.; Chicago, IL, USA). The EMG data were normalized at 100% during maximum voluntary contraction with Parafilm M®.

## Results

 Table 1 shows the comparison between the normalized EMG values of the clinical mandibular posture conditions and the masticatory cycles of the right masseter muscle (RM), left masseter muscle (LM) right temporalis muscle (RT) and left temporalis muscle (LT) for ZIG and CG.

**Table 1:**
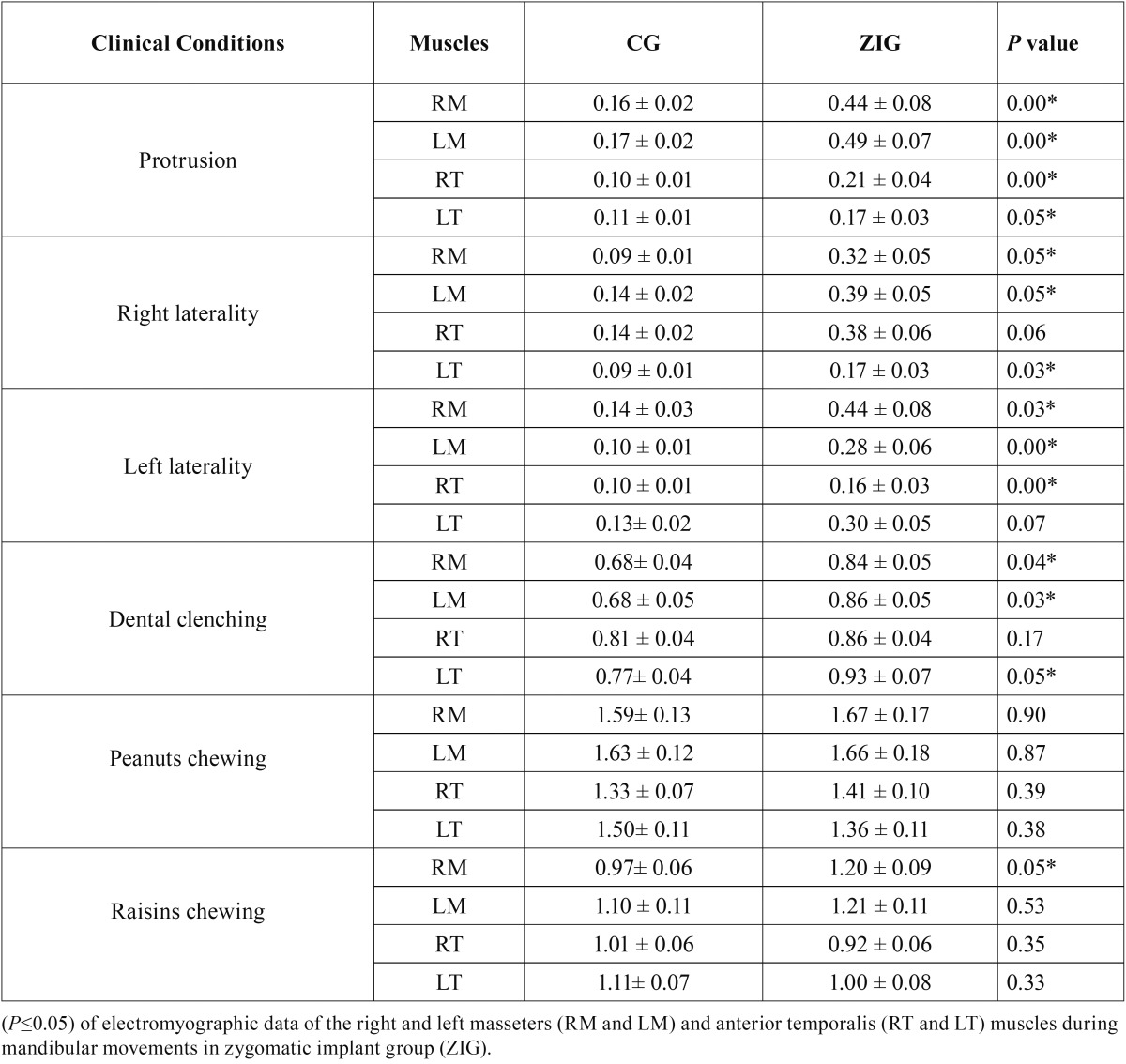
Mean, standard deviation (±) and statistical significance.

There was a statistically significant difference (*P* ≤ 0.05) in the EMG activity for protrusion (RM, LM, RT and LT), right laterality (except RT), left laterality (except LT), dental clenching (except RT) and for raisin chewing (RM).

The ZIG showed greater EMG activity during all mandibular posture conditions, when compared to CG. During mastication, both groups showed greater EMG activity for masseter muscles, when compared to temporalis muscles during chewing of hard and soft foods.

## Discussion

Advances in dental research promote the development of new technologies, which in turn, enable the manufacturing of implants, promoting osseointegration with faster and stronger bone formation (18). It contributes to the expansion of dental implant use and offers functional and aesthetic solutions to many clinical circumstances (19).

The procedures used in prosthetic rehabilitation of the oral cavity can cause functional and structural disturbances in the components of the stomatognathic system, which should work harmoniously during mastication (20). The occlusal stability generated by the use of implant-supported prosthesis may have significant effects on the muscle activity (21). Therefore, the use of EMG for the analysis of the masticatory muscles is extremely important. This diagnostic resource is non-invasive, safe and it helps to better understand the performance of these muscles in different clinical situations of speech, swallowing and chewing (22).

In the present study, EMG analysis was used to detect and record the activity of the muscle fibers, and to obtain additional information for the diagnosis, management and treatment of patients who need oral rehabilitation (23). Masticatory muscles activity was observed in patients rehabilitated with fixed prosthesis who were previously submitted to implant anchorage in the zygomatic bone. The EMG values were used to evaluate the masseter and temporalis-anterior muscles during the following clinical conditions: protrusion, laterality (left and right), maximal dental, maximal voluntary contraction, and mastication of hard and soft foods. The evaluation of masticatory muscles EMG activity has also become more and more useful to dentists as it adds to the knowledge of muscle performance during regulating reflex movements and in the changes to muscle pattern.

Considering that the EMG analysis involves many sources of biological and technical variability, the normalization of data was necessary and essential to determine the activity of the masseter and temporalis muscles in different clinical postural conditions (24). Special attention was given to the inclusion of individuals who have been wearing definitive prosthesis for at least six months. This is the necessary interval for anyone to show satisfactory chewing ability after the restoration was inserted (24).

ZIG group showed greater EMG activity when compared to control group. This finding can be explained by the stimulus sensation caused the presence of implants and the prosthesis base, although there were no periodontal nerve impulses. It is known that periodontal receptors play a significant role in the stomatognathic system, transferring afferent information from the tooth to the central nervous system and offering protection to the system itself (25).

During the mandibular posture condition and habitual chewing, the masseter muscles showed greater EMG activity than the temporalis muscles. This is expected in individuals who exhibit a normal activity of the stomatognathic system. The masseter is referred to as the strongest muscle based on the exertion of pressure during mastication (26). In contrast, the temporalis muscle is more associated with speed, and the first to contract during depression and elevation movements. It controls both retraction and elevation movements, and its action is less intense during mastication (27). A possible occlusion stability generated by the fixed prosthesis anchored in the zygomatic implant may improve the balance of the stomatognathic system, showing specific features that establish the masticatory stability through greater activation of the masseter muscles, compared to temporalis muscles (28).

Commonly, clinical parameter of mastication is used in oral rehabilitation for an accurate occlusal analysis in cases that include prosthetic devices, implants and temporomandibular disorders (29). Muscle activity levels are controlled by the sensory receptors and the central nervous system. Alterations in the occlusion-functional balance, due to the absence of teeth, cause severe changes in the masticatory muscle activity with direct effects on chewing (5). With the frequency records of the EMG signal linear envelope, it is possible to recognize when and how a muscle is activated or not. Then it is also possible to determine and establish the coordination of different muscles involved in the movement, as well as signaling some possible changes in masticatory efficiency (30).

There is no scientific evidence in the literature on the functional effects of the zygomatic implant technique discussed in this study, which poses a limit to the direct comparison of our results. The results of this study showed increased EMG activity of the masseter muscle during raisin chewing in subjects with implant-supported prostheses using the zygomatic implant system. Signif-icant differences were also observed for both masseter muscles (right and left) during all excursion movements and dental clenching. These results suggest that, muscle fiber recruitment in individuals rehabilitated via invasive surgical techniques can increase the myoelectric activity to perform the same masticatory patterns of a normal system (8).

Further studies are necessary to observe the behavior patterns of the masticatory muscles in individuals submitted to treatment with zygomatic implant anchorage, as well as the time of use, adaptation and stability of the prosthesis. Prosthetic rehabilitation is commonly used in dentistry (30); therefore, research on the topic is of paramount importance to achieve the correct muscle behavior pattern for the proposed treatment.

## Conclusions

Based on the analysis performed in the present study, it is possible to conclude that implant-supported prosthesis (anchorage in the zygomatic bone) offers unusual stimulus to EMG activity of the masseter and temporalis muscles when compared to fully dentate individuals.
